# Variable embodiment of stance-taking and footing in simultaneous interpreting

**DOI:** 10.3389/fpsyg.2024.1429232

**Published:** 2024-07-05

**Authors:** Alan Cienki

**Affiliations:** Department of Language, Literature, and Communication, Faculty of Humanities, Vrije Universiteit Amsterdam, Amsterdam, Netherlands

**Keywords:** stance, footing, gesture, simultaneous interpreter, pragmatic, self-adapter

## Abstract

Previous research has argued that consecutive interpreters constitute laminated speakers in the sense that they engage with different kinds of footing at once, representing another’s point of view through their words in another language. These multiple roles also play out in their gesturing, as they sometimes indicate deictically who is the source of the ideas and stances they are expressing (the principal). Simultaneous interpreters, though, often work in an interpreting booth; they are often not seen by the audience, yet many of them gesture, sometimes frequently. How are simultaneous interpreters using gesture in relation to stance-taking and footing? We consider the case of simultaneous interpreters rendering popular science lectures between (both to and from) Russian (their L1) and either English or German (their L2). Though only hearing the audio of the lectures, the interpreters produced many gestures, which were analyzed for their function. Some representational and deictic gestures appeared to clearly involve the interpreter as the principal (writing numbers with one’s finger to help remember them or pointing to two places on the desk to keep track of two different quantities mentioned). Other representational and deictic gestures are ambiguous as to whether they are enacting what the interpreter may have imagined what the lecturer did or whether they arose out of the interpreter’s own thinking for speaking (e.g., tracing the form of a bird being mentioned or pointing to an empty space when the lecturer was referring to a graph). Pragmatic gestures, showing one’s stance toward the topic of the talk, were the most ambiguous as to the footing, reflecting how the interpreter may be engaged in fictive interaction with their imagined audience. Self-adapters, however, more clearly involve the interpreter as the principal, as such actions are known to support cognitive focussing and self-soothing. In sum, we see varying degrees of clarity as to whose stance and principal footing simultaneous interpreters are expressing bodily as laminated speakers. The variable ambiguity can be attributed to the nature of gesture as a semiotic system, the functions of which are more often dependent on co-occurring speech than vice versa.

## 1 Introduction and theoretical background

### 1.1 Interpreting, gesturing, and thinking for speaking

If we reflect on the professional activity of simultaneous interpreting between two spoken languages, it is actually a very unusual activity. Interpreters are speaking while listening, and their speaking is in a different language than that being listened to. What the interpreters are speaking about does not concern their own ideas, opinions, or feelings, but those of someone else. Furthermore, simultaneous interpreting of spoken languages as it is usually performed in a conference setting or during a lecture often involves the interpreter being located out of view of the people being communicated to, i.e., those who are hearing the interpreter. Simultaneous interpreters are often seated in a booth, usually in the back of the room, and speak into a microphone so that they can be heard by those in the room equipped with headsets used for this purpose.

Given these unusual factors of this form of communication, it might be surprising to learn that many interpreters are gesturing with their hands while interpreting, even though there is no one viewing them (Stachowiak-Szymczak, 2019; Cienki and Iriskhanova, 2020; Martín de León and Fernández Santana, 2021). It is not unusual for people to gesture while speaking even when they cannot be seen by their interlocutor, as we know from the phenomenon of people gesturing while talking on the telephone. The gesturing in such cases has been argued to be an inherent part of the role of visual demonstration in dialogue, even when one cannot be seen by one’s interlocutor (Bavelas et al., 2008). The gesturing can also be seen as tied to processes of conceptualization (Kita et al., 2017), which includes processes of thinking for speaking. Specifically, Slobin (1987, 1996) claims that there is a special form of thought, thinking-for-speaking, that is mobilized when expressing one’s ideas verbally. It needs to adapt to the lexical and grammatical options that are available in the language being used in the moment. McNeill (1992 and elsewhere) calls the smallest unit of thought that has the capacity to grow into an utterance the “growth point.” When engaged in spontaneous talk, growth points successively develop and unfurl into speech and sometimes also into gesture in a dialectic process, whereby the lexical and grammatical forms afforded by the given language and the cultural constraints on the gestures influence each other in the microgenetic processes of their expression (McNeill, 1992, ch. 8). “Gesture contributes material carriers to thinking-for-speaking,” as McNeill and Duncan (2000, p. 157) argue.

Note, though, that the context in which simultaneous interpreters work, rendering others’ ideas in another language in a monologic fashion, is quite different from that of dialogic interaction. Furthermore, the kind of thinking involved in simultaneous interpreting is quite different from that involved in developing and expressing one’s own ideas. Not only is their speech not about their own ideas, but simultaneous interpreters’ later recall of what they heard while interpreting is significantly worse than it is when listening and not interpreting (Darò and Fabbro, 1994). This is in line with the view that interpreting need not involve (and most likely often does not involve) full semantic processing as much as a shallower form of lexical access (Gernsbacher and Schlesinger, 1997). Simultaneous interpreting is also an unusual form of communication in that the interpreters are not to express their own stance toward what they are saying (Setton and Dawrant, 2016, p. 344). This is something explicitly taught in interpreter training; for example, the “Practical Guide for Professional Conference Interpreters” of the International Association of Conference Interpreters (AIIC) states, “the interpreter must never betray any personal reaction to the speech, be it skepticism, disagreement, or just boredom.” Instead, the interpreter’s loyalty is owed to the speaker “and to the communicative intent that the speaker wishes to realize, whatever the speaker’s position or point of view” (AIIC, 2004/2012).

### 1.2 Stance-taking and footing

Stance-taking (Biber and Finegan, 1988, 1989) involves different aspects of the speaker’s attitudes toward their message, e.g., the degree of certainty about what one is communicating (epistemic stance), the importance of the information and the degree to which it is in focus (relevance stance), or one’s affectual relation to what is being communicated. In interaction, people can express their stance not only verbally, but also through visible behavior of different kinds, e.g., through their bodily posture (e.g., pulling one’s torso back when disagreeing with an interlocutor), as a form of physical stance-taking; facial expressions (e.g., raised eyebrows questioning another’s claims); and manual gestures. The role of the latter has been explored in a number of studies, from Kendon’s (1995, 2004, 2017) consideration of the functions of pragmatic gestures to the work on what Bressem and Müller (2014) and colleagues (e.g., Ladewig, 2014) have called “recurrent gestures.” Among the German speakers focused on in the latter studies, these include, for example, a palm-up open hand with a clockwise rotation to indicate vagueness or uncertainty, a brushing away movement for negative assessment, and a stretched index finger held upward to mark attention to what is being said (Bressem and Müller, 2014). Many of the families of gestures (Kendon, 2004) that these examples belong to are also recognized in other European cultures [e.g., French (Calbris, 1990), Italian (Poggi, 2014), and Russian (Grishina, 2017)], and we are just beginning to learn about other types of recurrent gestures in non-European cultures, e.g., Chinese (Harrison, 2021), Hausa (Will, 2022).

In addition to stance, there is the role that one has in interaction, which encompasses stance-taking. This is what Goffman (1981) characterized as footing. Goffman (1981, p. 128) notes that with footing, a “Participant’s alignment, or set, or stance, or posture, or projected self is somehow at issue.” If we take the case of spoken interaction, the footing most immediately related to the usage event (Langacker, 1988) of speaking is that of the *animator*, the role of being the person talking. Goffman (1981, p. 144) puts it plainly as the role of being “the sounding box in use” from which the utterances come. In some cases, though, the words being uttered might have been pre-determined and selected by someone else, as when a politician reads a speech written by a speechwriter. Goffman (1981) calls this role that of the *author*, the agent “who has selected the sentiments that are being expressed and the words in which they are encoded” (*ibid.*). Beyond that, one or more people might be responsible for the content of the words being uttered in terms of having epistemic authority over them. Goffman calls this footing that of the *principal*, the party “whose position is established by the words that are spoken, someone whose beliefs have been told” (*ibid.*). We can say that the principal is the one whose stance is expressed. Returning to the example of a politician delivering a speech, the party that Goffman refers to might be the political party that the politician represents.

### 1.3 Interpreters as laminated speakers

The various kinds of footing are not mutually exclusive. A leader of a political party who writes and delivers his or her own speech embodies all three roles. In other contexts, we may variably be taking on one or more forms of footing. Goffman (1981) observes that conversation (p. 154) and, more generally, experience itself (p. 156) are, as he calls it, laminated at various times, involving more than one kind of footing. Goodwin and Goodwin (2004) extend this to say that what Goffman presents is an “analytically powerful model of a laminated speaker” (p. 223) in terms of the different kinds of possible footing one may inhabit.

Considering the case of consecutive interpreters (dialogue interpreting), i.e., who render speakers’ utterances after they have produced them, Vranjes and Brône (2021) point out that such interpreters are laminated speakers of a special sort. The interpreter is the animator of the words they are speaking, but the person whose words they are interpreting is the principal. However, who is the “author” of the interpreter’s words? Vranjes and Brône argue that the author is both the interpreter and the one whose utterances are being interpreted, and in this sense, the interpreter is a laminated speaker. But consecutive interpreters negotiate the interaction not only verbally between the people who speak two different languages but also co-verbally, using eye gaze direction, head movements, and deictic hand gestures. They note (Vranjes and Brône, 2021, p. 97), “Our analysis reveals that interpreters have a repertoire of multimodal resources at their disposal to layer their utterances and draw attention to the principal while rendering the talk.”

### 1.4 The research questions of the present study

In simultaneous interpreting, though, as discussed above, the interpreter is normally not physically in the interactional space with the speaker of the source text and the audience hearing the interpreter’s renderings in the target language, and in fact is usually not even visible. Yet, such interpreters are gesturing in many cases. This gives rise to several questions concerning the relations between gesture, footing, and stance, which will be examined here, specifically:

1) How does the use of different functions of gesture during simultaneous interpreting relate to the role of the interpreter as a laminated speaker? In what ways is this similar to or different from the situation with consecutive interpreters, as discussed in Vranjes and Brône (2021)?
2) How are simultaneous interpreters using gesture in relation to stance-taking, as discussed above in section 2.1?

3a) Whose stance (which footing?) is being expressed in any given interpreter’s gestures moment by moment in the process of rendering the original speaker’s utterances?
3b) Can this even be determined in the context and conditions of simultaneous interpreting?

As we will see below, considering the different functions of gestures and viewing them through the lenses of footing and stance-taking can help us gain further insights into what may be involved in simultaneous interpreters’ processes of thinking for speaking.

## 2 Method of data collection

Forty nine simultaneous interpreters were involved in the study conducted at a university in Moscow between 2019 and 2021. All were native speakers of Russian (Russian as L1), residing in Russia, mostly in Moscow. Twenty nine of them (13 female) (average age = 33 years old) were experienced interpreters working between Russian and English (average 9 years’ experience in interpreting) and 20 (7 female) (average age = 33) between Russian and German (average 10 years’ experience). The materials they were asked to interpret in each case were two ten-minute excerpts from science lectures originally delivered to audiences of laypeople: one in Russian which they were asked to interpret into their main second language or L2 (be that English or German) and one in their L2 which they interpreted into Russian. All of the lectures concerned issues around biodiversity on the planet and the extinction of species of animals. The Russian lecture in each case, from the popular science website PostNauka, addressed the question, “Is there a threat of a sixth mass extinction of species?”^1^ The lecture in standard British English, a TEDx Talk, was on “Mass extinctions and the future of life on Earth.”^2^ The German lecture, from the ARD television’s Mediathek website, was entitled “The end of evolution.”^3^

The interpreters were provided with vocabulary lists several days in advance with discipline-specific terminology from the videos and suggested translations into Russian and the L2, as appropriate. However, they were not allowed to bring any materials (such as paper, pens, or mobile phones) with them into the interpreting booth in which they were recorded. The reason for this is that we^4^ were interested in how they handled the cognitive load of the interpreting sessions unencumbered by external tools. This allowed for a uniform condition across participants (i.e., no variation in terms of what external resources they might use) which also afforded studying their free-handed gestural behavior.

Written informed consent was obtained from all the participants in advance, and all interpreters were assigned participant numbers to anonymize reference to them. They were not informed in advance that our study was focused on gesture use and initially were only told that we were interested in analyzing interpreting behavior. The sessions were conducted in an interpreting booth in an otherwise empty classroom used for training interpreters. The interpreter sat on a chair with no armrests at the small desk built into the interpreting booth. Three video cameras recorded each interpreting session. A Sony HRX-NX30P video camera with a Sony ECM-XM1 directed microphone attached was placed on a tripod to the side behind the interpreter, pointed downward to provide an over-the-shoulder view of the interpreter’s hands on the desk. On the far edge of the desk, a small GoPro HERO3+ Silver camera was placed facing the interpreter. This recording angle gave a close-up view of the interpreter’s hands and face. In addition, interpreters wore Tobii eye-tracking glasses while performing the task, which provided a view through the glasses of where interpreters were looking, but this viewpoint is not of concern for the present analysis.

Each participant interpreted a 10-min segment from the talk in Russian into their L2 and from the talk in their L2 into Russian. The order of the tasks was randomly varied per participant. Importantly, interpreters only heard the audio of the lectures, played to them via headphones attached to a laptop placed out of view on a small stool to the side of their interpreting desk. They did not see the video of the lectures (the video was not even played off of the laptop—just an audio file of the lecture was used). This was done so that the interpreters could not see the actions of the speaker and so would not be influenced by their gestures (they could not copy them). Each time, a one-minute warm-up portion of the audio that preceded the upcoming 10-min portion was played in order to allow the researchers to adjust the audio to the interpreter’s wishes and to allow the interpreter to get used to the lecturer’s voice and speaking rate. Then the researcher began the 10-min portion of the lecture, closed the door of the interpreting booth, and moved to a part of the classroom out of view of the interpreter (since the interpreter’s desk faces a large glass window in the door, looking into the empty classroom).

After the interpreting sessions, participants were debriefed about the study and they were allowed to choose how we could use the recordings of their interpreting, with permission options ranging from the maximum (being allowed to post audio or video clips of their sessions on academic websites) to medium (permission to post or publish screenshots of them from the videos) to the minimum (permission to only publish drawings of their gestures).

## 3 Methods of analysis

The recordings of the two ten-minute sessions from each of the 49 interpreters results in 16 h and 20 min of data. For practical reasons, 2 min were taken from each of the 98 videos for detailed analysis—one near the beginning of the session, after the interpreter had gotten into the flow of the task (minute 3:00–3:59) and 1 min later in the session (minute 8:00–8:59). The videos were imported into the ELAN^5^ software (Sloetjes and Wittenburg, 2008) for analysis. The speech was transcribed using standard orthography for the given language. Gesture units were annotated with each including any preparatory phase, stroke phase, and post-stroke hold, if there was one (following Kendon, 2004).

Gesture were coded for functions in the context of the interpreter’s speech using a system adapted from those used in Müller (1998), Cienki (2010), and Bressem et al. (2013). Though gestures are often multifunctional in nature (viz. Kok et al., 2016), we aimed to identify the primary function of each gesture, as described in brief below. In cases where interpreters were gesturing with two hands at once, and the two hands were seen to be realizing different functions, we coded the function of the dominant hand, that being the hand with which the interpreter gestured the most in the recording. The resultant code book consisted of the following categories of gesture use: representational, deictic, pragmatic, and as an adapter.^6^ The following descriptions are abbreviated versions of those from the code book.

•The representational function is accomplished through depiction of some content of the speech. This was assessed if any one of five modes of representation (adapted from Müller, 1998, 2014a) was used. These entail either *acting* as it might when performing an action involving an object (such as moving one’s fingers up and down as if typing on a keyboard); moving one’s open hands as if touching the surface of an object (so-called *molding*); keeping one or both of one’s open hands in a position with palms facing each other or with the palm up as if *holding* an object; *tracing* a shape or line with one’s fingertip(s); or using the hand to as if become an object, as when one’s index and middle fingers are extended straight and *embody* a pair of scissors by separating and closing together again.

•The deictic function is accomplished through one or more extended fingers being used to point in a direction or to touch a surface (such as the interpreter’s desk) to identify a spatial location, an imagined referent, or a moment in time.

•Pragmatic functions include various types. These include performative functions (showing whether one is posing a question, making a denial or an offer, etc.), parsing functions (e.g., indicating topicalization or commenting via one’s utterance), and modal functions (including negation, intensification, evaluation, etc.) (Kendon, 2004, pp. 281–282). What Kendon calls modal functions involve showing one’s attitude toward the current topic of the talk. Here we see the expression of epistemic stance, relevance stance, and affectual or attitudinal stance. Bressem and Müller (2014), for example, show how for German speakers a wavering open hand can express uncertainty or doubt (epistemic stance), beats can emphasize words being spoken (relevance stance), or an open hand, palm facing down or away from the speaker and moving laterally can express dismissal or rejection (attitudinal stance). Whereas the representational function relates directly to the semantics of the speech (occurring with or beginning just after the start of the gesture unit), and therefore such gestures may be unique in form, pragmatic gestures occur across many contexts, with similar groups of forms expressing related functions (thus the name “recurrent gestures”). Many gestures serve both representational and pragmatic functions, such as metaphorically holding a referent as if it were an object in the hands while also performatively offering the imagined referent to the addressee; therefore, in our study, the category of pragmatic function was reserved for cases when it was clear that the primary function was not that of representation.

•Adaptive functions, as discussed in Ekman and Friesen (1969) involve either self-adapters or other-adapters. Self-adapters are inwardly oriented movements, involving self-touching of some kind. Other-adapters are externally oriented and entail touching some object, such as rubbing the desk. Adapters may consist of discrete, one-off actions, such as quickly scratching oneself or pushing back one’s hair, or sustained actions, such as rubbing one’s fingers together for an extended time. Note that this category of adaptive functions is excluded from many gesture studies because of researchers’ focus on referential and pragmatic functions of gesture. However, in the present study, we included them because of their prominent role, as discussed below.

The video analysis was performed by nine researchers in the project, who worked in three teams of three members each. The videos were first divided among the three teams for the gesture annotation and function coding. Consensus checks on the coding were conducted within each team. The ELAN files with the videos were then exchanged with other teams who checked if they agreed with what were annotated as gesture units and performed an independent coding of the functions of the annotated gestures. Discrepancies between annotations and coding were then discussed and resolved at regular meetings of the entire research group. The method here was inspired by that described in Stelma and Cameron (2007); in their case, it concerned the coding of intonation units in transcribed talk. The method they used, and that they recommend, involved annotation and re-annotation of a transcript by a given individual, with refinements over time based on consultation with other experienced researchers. In our case, however, the individual annotations were not created and coded for function by one individual, but rather were done independently by the three members of one team and three members of another team (thus six coders) to check if the consensus within each of the two teams matched. Final resolution of any remaining problem cases took place through discussion with all 10 project members. Any amendments to the code book resulting from clarifications coming out of the discussions of the cross-checking were then applied across all the videos. This procedure was followed for all the coding, not just with a small percentage of the data, as is often the case for a cross-check of inter-annotator agreement.

## 4 Results

As reported in more detail below, the vast majority of the 3,719 gestures produced over all of the interpreting sessions either had a primarily pragmatic function or (self-)adaptive function. Far fewer gestures served a representational or deictic function; below we will consider these two categories together as constituting different types of referential function. We will consider the possible reasons for this distribution of gesture functions in relation to how they relate to footing.

If we consider Goffman’s account of footing in talk and translate it to gesture, we can say the following. The animator can be seen as the one moving who is producing the gestures. Therefore an interpreter gesturing while interpreting is the animator of the gestures they are producing. The principal can be said to be the party to whose position, stance, and/or beliefs the gestures attest. Therefore, the question to be discussed below is: whose stance do the interpreters’ gestures reflect? Their own, or the imagined stance of the original lecturer being interpreted? Finally, there is the footing of the author. We could say that that is the agent who puts together, composes, or scripts the gestures that are produced. This would make sense in the context of a play or a movie, for example, where the director and/or the actor decide in advance what gestures will be produced when, or for a public speaker who has been advised by a communication consultant on how to gesture. However, in the context of simultaneous interpreters, this footing is not relevant. The only way in which it might be relevant is if interpreters were trained to gesture in certain ways. In fact, the interpreting tradition in the cultural context considered here (at the university in Russia where these interpreters were trained) often advises simultaneous interpreters to sit still at their desk, usually with hands folded, not gesturing, so as not to attract attention to themselves. Therefore, in the analysis of the results below, we will focus on the footing of the principal, giving special attention to the questions mentioned at the end of Section 1 above.

### 4.1 Gestures with referential functions

#### 4.1.1 Representational gestures

Only 6% (*N* = 216) of the 3,719 gestures in total were representational in function. This amount hardly differed in relation to the direction of interpreting between L1 and L2 (L1 to L2 7%, L2 to L1 6%). Examples of representational gestures included instances where the interpreter was rendering a number, and while doing so, traced the written number on the desk with their finger, as if their finger were a pencil writing the number. This helped one interpreter, for example, who was rendering a phrase from Russian into German: having heard in Russian “*ot soroka tysjač do semidesjati tysjač vidov v god*” (‘from forty thousand to seventy thousand species per year’) and saying in German, “*mm […] von… vierzig [bis ehm*… *seib]zig Tausend Arten per Jahr*” (‘mm […] from… forty [to uhm… seven]ty thousand species per year’). (Square brackets will be used to indicate the speech or pauses co-occurring with the stroke phase of each gesture. The length of pauses relative to the speaker’s speech rate is indicated by either two dots for a shorter pause or three dots or more for a longer pause.) With her two hands resting on the desk, the interpreter moves her right hand palm down and with the middle finger she “writes” the numbers 4 and 7 on the desk at the first and second moments enclosed in square brackets in the phrase transcribed above. This kind of representation of the content being interpreted appears to be a means for the interpreter to keep track of the information to be rendered. It is well known that numbers can present a challenge in simultaneous interpreting (Mazza, 2001; Pellatt, 2006) because the information they convey is not predictable in the way that, for example, fixed phrases in a language are. Indeed, it is common practice to write down numbers during simultaneous interpreting so as not to forget them. In this respect, it seems clear that the principal behind the gestures in this case is the interpreter herself; that is: it is far less plausible that the interpreter may have imagined the original lecturer himself writing down the numbers 4 and 7 when he was uttering “forty thousand” and “seventy thousand” during his lecture.

**Figure 1:**
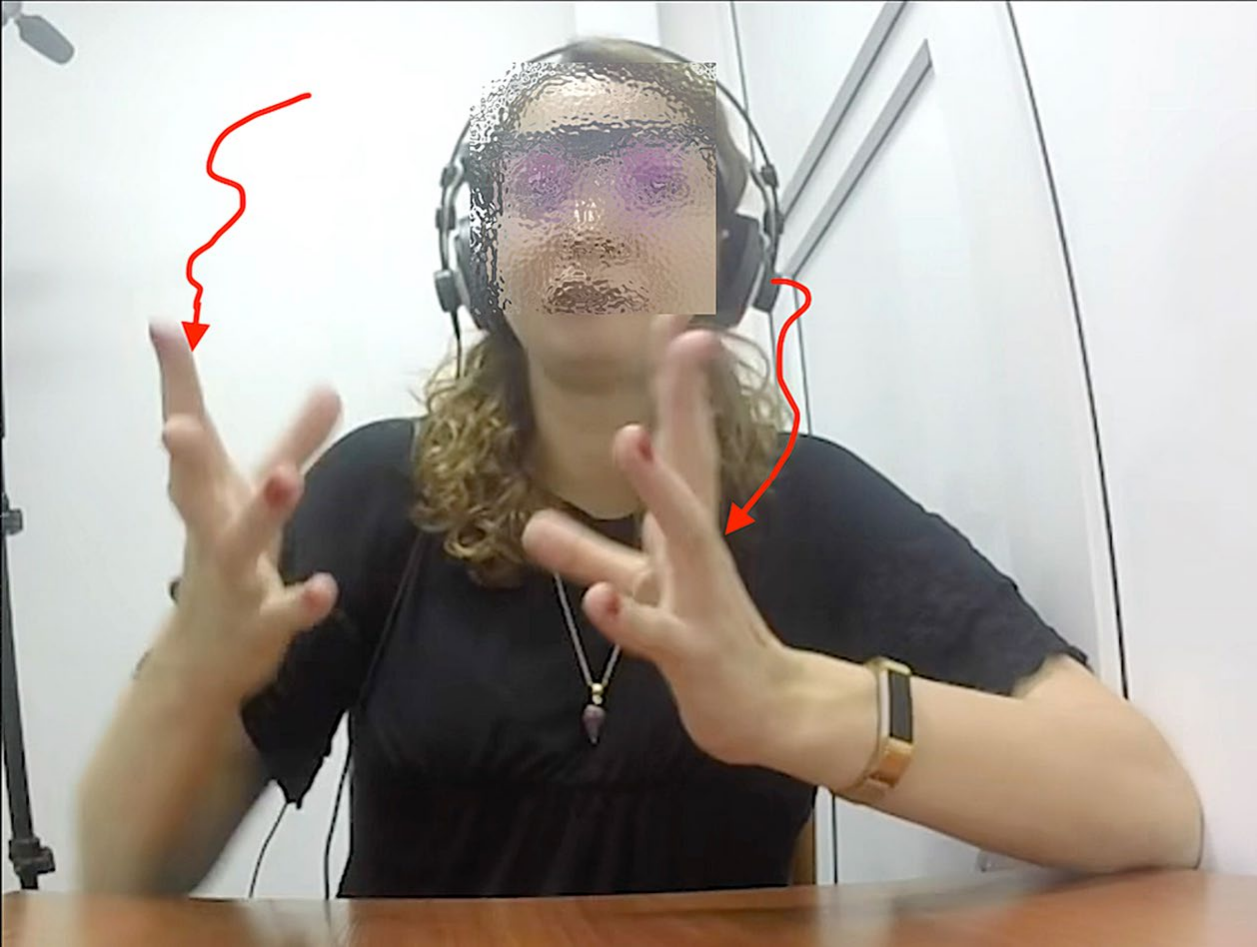
A molding representational gesture while saying in Russian, “*i my vse znaem, [kak vygljadel dodo] v ètoj knižke*” (‘and we all know [what the dodo looked like] in that book’).

In another case, the interpreter renders a phrase about the famously extinct dodo bird, saying in Russian, “*i my vse znaem, [kak vygljadel dodo] v ètoj knižke*” (‘and we all know [what the dodo looked like] in that book’). The gesture stroke co-occurring with the bracketed phrase involved holding up both open hands facing each other and quickly moving them downward in a wobbly path, as indicated in Figure 1.

This qualifies as a molding gesture, as if touching the surface of a medium-sized object that is somewhat taller than it is wide—just as the image of the extinct bird is often shown, in a standing position.^7^ Here the principal of the gesture could be the interpreter, based on her own thinking for speaking, but it is also not implausible to imagine the original lecturer perhaps gesturing a rough image of a medium-sized dodo-object in the air as he mentioned it. Given that the interpreters were only hearing the lecturer and not seeing him, it is possible that they might mentally simulate (Marghetis and Bergen, 2014) the gestural production of the speaker they were hearing. Perhaps both phenomena are possible at the same time—the interpreter gesturing the general shape and size of her mental image of the dodo, enacting what one might plausibly imagine the lecturer could have done. The ambiguity here reflects one way of understanding the laminated nature of interpreters in their task as speakers (Goodwin and Goodwin, 2004).

#### 4.1.2 Deictic gestures

**Figure 2:**
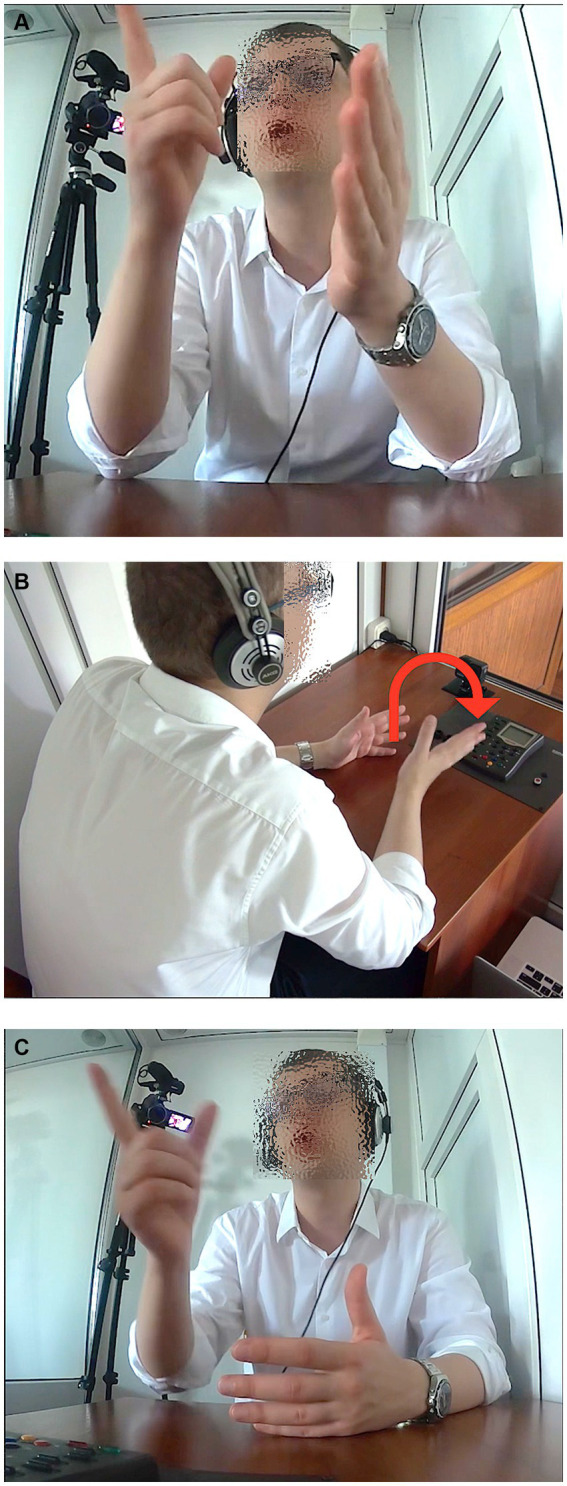
**(A)** A deictic gesture while saying in Russian, *“[vot tut], …vy vidite, naprimer…*” (‘[right here], … you see, for example…). **(B)** A deictic gesture while saying in Russian, “*u[tra]tu ploščadej doždevyx lesov*” (the [loss] of acreage of rain forests’). **(C)** A deictic gesture while saying in Russian, “*no ne [tol’]ko tam. V vostočnoj Azii èti processy takže otmečajutsja*” (‘but not [on]ly there. These processes have also been noted in East Asia’).

An even smaller amount of the gestures, namely 3% (*N* = 114), was deictic in function (L1 to L2 4%, L2 to L1 2%). One type of deixis observed was that of pointing gestures, with an extended index finger or flat hand. However, the pointing was not to physical referents in the interpreters’ surroundings; they were not talking about the place in which they were located while performing the task. Instead, in a few cases they pointed to a space off to the side when interpreting an utterance by the lecturer that made reference to a graph or map that he was showing. Again, the interpreter was not viewing a video of the speaker, and so had no information as to where the image being referred to was being shown (e.g., on which side of the speaker). In the example shown in Figure 2A, the speaker, interpreting the German lecture, says in Russian, *“[vot tut], …vy vidite, naprimer… u[tra]tu ploščadej doždevyx lesov*” (‘[right here], … you see, for example… the [loss] of acreage of rain forests’) and points to the upper right (Figure 2A), also directing his eye gaze there on “vot tut” (‘right here’) and also moves his right hand, fingers extended, in an arc to the right and makes a beat downward when saying “utratu” (‘loss’) (Figure 2B), with his hand almost touching the desk.^8^ Seconds later he says, “*no ne [tol’]ko tam. V vostočnoj Azii èti processy takže otmečajutsja*” (‘but not [on]ly there. These processes have also been noted in East Asia’) and again makes a pointing gesture to the upper right with his index finger (Figure 2C).

The pointing was from the imagined viewpoint of the lecturer, pointing to the imagined physical chart being cited. Here one can argue that the principal of the gesture is the lecturer (or lecturer as mentally simulated). The viewpoint (conceptually, and even physically, as the interpreter looks up to the space he is pointing to) of the lecturer is blended with the interpreter’s embodied rendering of it, as if the interpreter were pointing in place of the lecturer.

**Figure 3:**
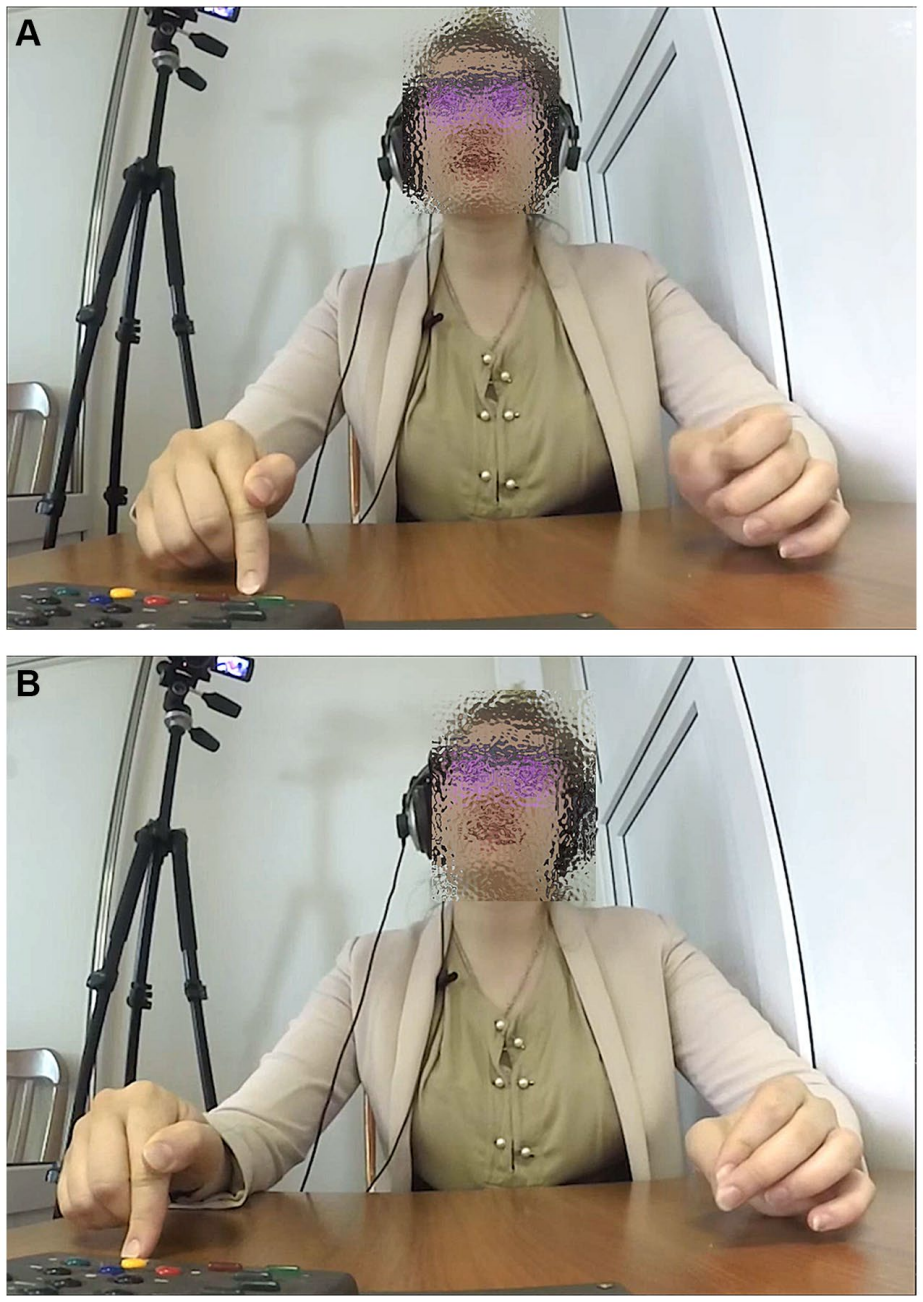
**(A)** A deictic gesture while saying in English, “*from th- [for]ty thousand*.” **(B)** A deictic gesture while saying in English, “*to [se]venty thousand… species per year*”.

One other type of deixis involved touching. In the same portion of the Russian lecture mentioned earlier, when two numbers were cited, the interpreter, in this case rendering the lecture in English, said, “*from th- [for]ty thousand to [se]venty thousand… species per year*.” During the bracketed syllables, she touched the desk in front of her in two different places, shown in Figures 3A,B, locating the amounts as points in space, metaphorically objectifying the quantities as locations. Interestingly, the second point, identifying a higher number, was laterally to the right of the first point deictically touched, as if on a number line.

As with the example of tracing the numbers on the desk, the gesture appears to be based on the interpreter as the principal, keeping track of the two quantities mentioned. It is also conceivable (though perhaps less plausible) that the interpreter may have gestured in this way based on an imagined (mentally simulated) anticipation of what the original speaker may have done when mentioning these numbers. This could constitute another example of the potential ambiguity of principal footing in the gesturing of the simultaneous interpreters.

Though the referential function of gesture (representation or deixis) constituted the smallest proportion of gestures used, it nevertheless raises some intriguing questions about the footing behind the interpreters’ gestures. The use of gestures with pragmatic functions, discussed in the next section, presents further puzzles when the issue of stance comes to the fore.

#### 4.1.3 Gestures with pragmatic functions

Forty four percent (*N* = 1,638) of the gestures were pragmatic in function. Here there was a small difference based on the direction of interpreting: L1 to L2 40%, L2 to L1 47%. Let us consider three examples of different kinds of pragmatic uses of gestures that were observed before considering how they might be interpreted in terms of footing. Though one specific instance of each will be described, each type was used by several of the interpreters.

**Figure 4:**
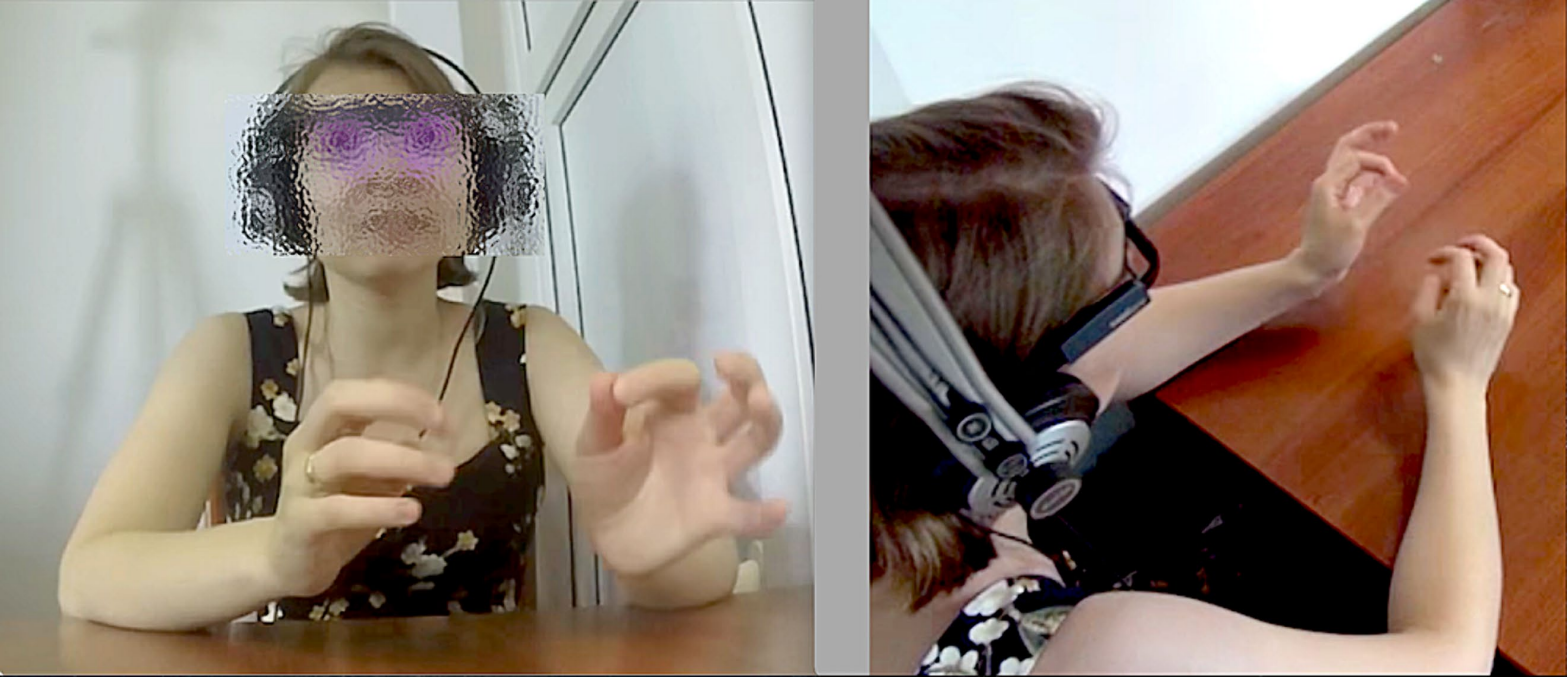
A pragmatic precision-grip gesture while saying in English, “<inbreath>… *[sev]en. [spe]cies [of] [birds]*”.

In one example of interpreting from Russian to English, the participant uttered the phrase “<inbreath>… *[sev]en… [spe]cies [of] [birds]*” (followed by the phrase “*are now extinct there*”) while holding her left hand in a position with the tips of the thumb and index finger touching, the fingers thus making a ring shape (even if not a perfect circle) while the other fingers were extended and slightly curved, as shown in Figure 4. She moved her hand down in a beat with the prosodic stress on each of the syllables marked in square brackets above.

This handshape, with the palm facing the central gesture space, as shown in Figure 4, is a variant of the ring gesture (Kendon, 2004, pp. 238–247; Müller, 2014b). It can be used by speakers (at least of the European languages studied to date in relation to gesture use) when making a precise point. Morris (2002, pp. 78–79) therefore refers to it as the “precision grip,” as the thumb and forefinger would be used to grasp and hold a tiny object (on this see also Calbris, 2011, pp. 21–22; Lempert, 2011). Therefore the use of this gesture can be related to both epistemic stance (showing the precise certainty of the information being uttered) and relevance stance (showing that this information is important, putting it in focus).

**Figure 5:**
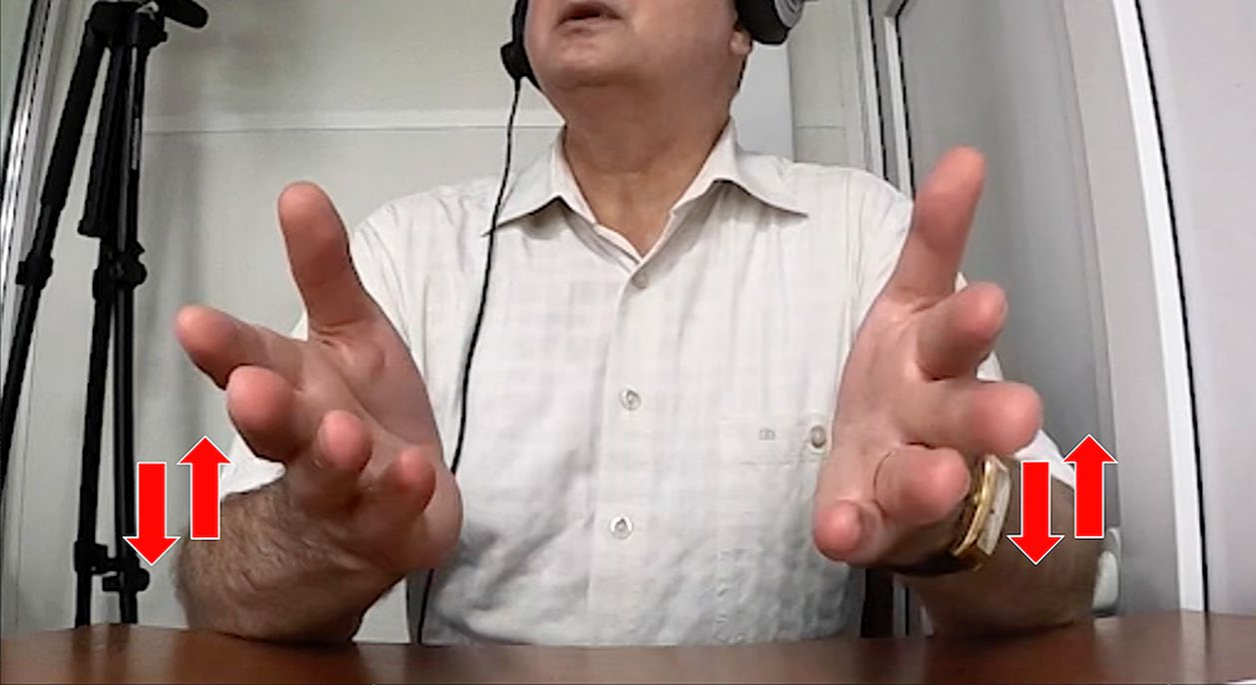
Pragmatic beat gestures while saying in German, “*áusstérben óder nícht*” (‘are dying out or not’).

In another example, the interpreter had referred to 90% of the species on Earth, after which he continued, interpreting from Russian to German, “*{wir können |nichts sag|en, ob sie [aus}sterben oder nicht], aber*” (‘{we can|not say anything| about whether they are [dy}ing out or not], but’). In addition to the square brackets [] indicating the words co-occurring with a manual gesture, the curly brackets {} indicate the phrase with which the interpreter rapidly shook his head with small movements back and forth, to the left and to the right, several times. The vertical pipes | | indicate the syllables on which he raised his right shoulder slightly, once on each syllable. During the phrase in square brackets, he turns out his two open hands, fingers outstretched, as shown in Figure 5, making beat movements downward on the four syllables marked here: “*áusstérben óder nícht*.”

The opening of the hands combined with the downward beats is similar to the palm-up gesture speakers may produce when presenting a point (Müller, 2004). Combined with the lateral headshaking and the shoulder lifts, we see three components of the complex enactment known in English as a shrug. Debras (2017), Jehoul et al. (2017), and Streeck (2009, ch. 8) discuss the function of the shrug in expressing a stance less committed to the information being uttered (what Debras and Cienki, 2012, refer to as “dis-stance”), and/or uncertainty, in terms of epistemic stance. Even the various individual components may relate more to the expression of particular aspects of the stance. Thus while the lateral headshake is known to express negative assessment in most European cultures (Harrison, 2014), Debras, (2017) notes that the raising of one shoulder more often expresses an affective stance (indifference or rejection), while the turning out (supination) of one or both hands correlates more with the attitudinal expression of incapacity to know or to take action. In the example considered here, the differential timing of the use of the different components shows a dynamic shift from negation (we cannot say whether these species will die out) to the attitudinal stance of admitting that we are incapable of knowing this.

**Figure 6:**
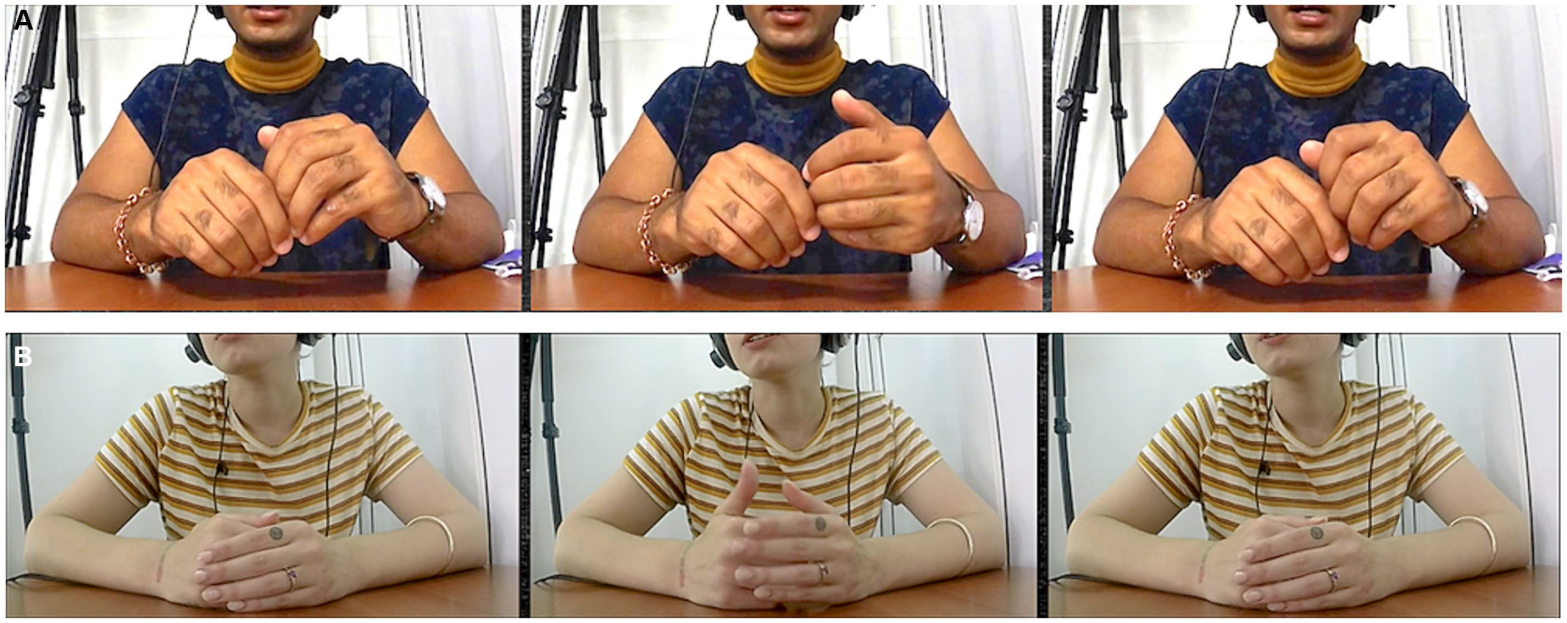
**(A)** A pragmatic small hand turn-out while saying in Russian, “*èto bylo [vygodno]*” (‘it was [advantageous]’). **(B)** A pragmatic lifting of the thumbs while saying in English, “*what [kind of] animals*”.

The third example encompasses a set of instances of mentioning a point accompanied by a small turn out of the hand (resulting from a small rotation of the forearm), outward and back in, as in Figure 6A, or even just an extension of one or more fingers of the hand and then a return back to the starting position. In some instances it simply involved a lifting of one or two thumbs if the hands were folded on the desk, as shown in Figure 6B.

These types of gestures have been analyzed (Cienki, 2021) as miniature variants of the palm-up open hand, well known as a gesture used when presenting a point (Müller, 2004; Cooperrider et al., 2018). They provide a minimally effortful way to show information status (relevance stance), indicating the point being uttered verbally as something to be taken into consideration.

In terms of the principal footing, the question we are left with is whether the use of these stance-taking gestures in the context of simultaneous interpreting (where the original speaker is not seen) derives from the imagined behavior of the original lecturer (the imagined stance of that speaker), or the interpreter’s own stance, or whether there might be other explanations. This issue will be considered further in the Discussion and Conclusions, below. It is interesting to remember, though, that the interpreters did not have any visible audience that they were speaking to. While it is true that they were being recorded, the larger camera was out of their view (behind them) and the other camera was a small, unobtrusive GoPro on the desk in front of them, as can be seen in Figure 2B. In addition, previous research (e.g., Mol et al., 2011) has shown that speakers do not produce more gestures simply when a camera is present or because of other people being in the room, but rather when they knew an addressee could see them, namely in settings where they could see their addressee’s eye gaze. Nevertheless, in the present study, many of the interpreters produced many gestures.

#### 4.1.4 Gestures with an adaptive function

**Figure 7:**
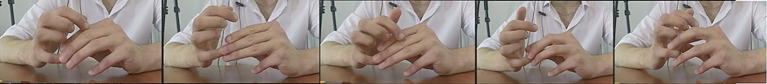
A sustained self-adapter, rubbing one’s finger while speaking (time elapsed: 8 s).

Of the gestures serving an adaptive function, the majority (44%, *N* = 1,636) were self-adapters, with a slight difference in the amount depending in the direction of interpreting (L1 to L2 46%, L2 to L1 42%). Only 3% (*N* = 115) were other-adapters (3% being the proportion for both L1 to L2 and L2 to L1 interpreting). Most of the self-adapters were sustained in nature: the position that many of the interpreters assumed, starting with their hands folded on the desk, afforded movements like rubbing one hand with the fingers of the other (see Figure 7), or moving one’s hand down to rub one’s arm. Sometimes this even took more extreme forms, involving pulling on one’s own skin.

In terms of principal footing, it does not seem likely, while engaged in the cognitively intense task of simultaneous interpreting, that the interpreter was picturing the original speaker making such small sustained movements while lecturing. It is much more plausible that such movements serve the interpreters’ own purposes of cognitive focussing, perhaps even self-soothing (Freedman, 1972) to relieve some of the stress of the task. Here the principal behind these movements is more clearly the interpreter. In this regard, we might say that the lamination discussed above comes apart momentarily when orienting inward, using self-adapters; the interpreter’s footing in such moments is less multifaceted than when engaged with outwardly-oriented gestures that might embody what the lecturers could have been doing as part of giving their talk.

## 5 Discussion and conclusion

We see that interpreters are laminated speakers in more ways than just in their use of speech (as Vranjes and Brône, 2021, point out). However, there are varying degrees of differentiation as to whose stance and principal footing they are expressing bodily.

On the verbal level, the principal of what the interpreter is uttering is clear: it is the speaker of the source text, in our case: the original lecturer being heard. Only rarely are interpreters the principal of the words they utter; this can occur momentarily when they correct what they said and add “Excuse me” or the like in the target language. Here the switch in footing is discrete (excusing themselves in that moment), sandwiched between the renderings for which the lecturer of the source text is clearly the principal; that is, they are not asking the hearer to excuse the original lecturer.

However, we have seen that it is often not possible to clearly determine the principal footing behind simultaneous interpreters’ gestures. This is quite different from the situation that Vranjes and Brône (2021) describe for consecutive interpreters, where the speakers of the source text are present as interactants along with the interpreter and the audience of the interpretation. In that context, eye gaze direction, head nods, and manual pointing gestures are sometimes used to indicate that the principal of an interpreted utterance is not the interperpreter him/herself but the original speaker, who is visibly present. Vranjes and Brône point out that the verbal attribution of the principal can sometimes be confusing for listeners during interpreting, given interpreters’ convention of maintaining the original speaker’s use of the first-person pronoun (i.e., it can be confusing that the “I” used the interpreter means someone else). But gesture use in consecutive dialogue interpreting can disambiguate that the interpreter, as the animator and author of the interpreted utterance, is not the principal. The difficulty in determining the principal as displayed in simultaneous interpreters’ gestures is partly due to the fact that all of the interactants (speaker of the source text, interpreter, and audience of the interpretation in the target language) are not sharing attention in the same space where they can all see each other. There is not one framework for deixis to operate in, for example.

Ascertaining the principal behind the pragmatic gestures observed in this study presents a particular puzzle, as noted earlier, and relates to the condition of the participants (speaker, interpreter, listener) not sharing one interactive space, visually accessible to all. Though some of the pragmatic uses of gesture discussed here (components of the shrug and hand turnouts as presentation gestures) were considered interactional functions by Bavelas et al. (1992), the interaction here is fictive, in the sense of Pascual (2002, 2014). Pascual builds on Talmy’s notion of fictivity, which refers to “the imaginal capacity of cognition” (Talmy, 2000, p. 100) and an “as if” state of affairs. Therefore, fictive interaction can be distinguished from factual (objectively verifiable interaction with someone else in real time) and from fictional or fictitious [interaction “conceptualized as occurring in a fantasy world or even in a hypothetical or counterfactual scenario” (Pascual, 2006, p. 384)]. So while the fictive interaction in our study could theoretically be conceived of as mentally simulated interaction (e.g., with an imagined audience), the level of cognitive load that simultaneous interpreters are already handling makes this explanation less plausible.

A more tenable explanation for such use of pragmatic gestures might be that they are so ingrained as part of the process of spoken interaction, at least among adult speakers, that interpreters cannot help but produce them when they would themselves engage in stance-taking when presenting the points that they are uttering. This might explain the slightly greater proportion of pragmatic gestures when interpreting from L2 to L1. In one’s native language and culture, one’s routines for engaging in talk in interaction are more ingrained; one has a handy repertoire of recurrent gestures that one can resort to. (We can contrast this with the slightly higher proportion of self-adapters found in the interpreting from L1 to L2.) The fact of gesture use even in contexts in which no interlocutor is present or visible highlights the inherently intersubjective nature of language. As Cuffari (2024, p. 611) captures it, “Gesturing and intersubjectivity are multifaceted yet reciprocally informing phenomena that presuppose each other.” However, Hostetter and Alibali (2008) point out that speakers do not gesture with every utterance, but rather they do so when the motivation to gesture reaches and exceeds a certain threshold. The threshold can be higher or lower depending on a complex of factors, including the individual speaker’s habits, the cognitive effort they are exerting in the moment, the discourse context (what was being talked about previously), the social context (more formal versus more relaxed), etc. Given the varying strength that the various factors may have in the present context, it would explain the wide variation across the interpreters (individual variation) in their use not only of pragmatic gestures but of gestures in general.

In addition, the difficulty in terms of attribution of the principal behind gestures in this context is partly a factor of the nature of gesture in general as a semiotic system. Gesture is arguably more dependent on speech and contextual information in most contexts than speech is dependent on gesture (viz. Kibrik and Molchanova, 2013). Gesture is generally underspecified in form in relation to function. If we take deictic gestures, for example, it is well known that someone observing them is dependent on context for determining the target that the gesturer may have intended with their pointing (Kendon, 2004, ch. 11; Kita, 2003; Talmy, 2017). If we think of representational gestures, the depiction involved is always metonymic (synechdocal), iconically showing only a part of some referent (Mittelberg and Waugh, 2009, 2014; Müller, 2014a). That is part of how the modes of representation function: they provide schematic imagery. Some of the representation may be based on schematizations of everyday actions, such as put in, take out, sit, run, etc., what Zlatev (2005, 2007) has called mimetic schemas (see also Cienki, 2013; Zlatev, 2014). Other instances of gesture use may draw upon even more general patterns in our everyday experience, what Johnson (1987) has discussed as image schemas, such as containment, balance, or path (Cienki, 2005). Turning to pragmatic gestures, they are sometimes produced in less effortful forms; speakers might not produce the full compound enactment of a shrug, mentioned earlier, but just a small part of it, with less effort. The presentation gesture—the archetype of which might be the magician presenting the result of a trick and exclaiming “Ta daa!” with a full turning out of a palm-up open hand—is more often produced in everyday conversation in reduced forms, with the hand not fully turned palm up, and perhaps with just a finger extended outward (Cienki, 2021). In these ways, schematic instantiations of pragmatic gestures are all that speakers produce in many instances. As Mark Turner (personal communication, cited in Cienki, 2017) phrased it, if we consider any expression, be it verbal or gestural, “the product is a given precipitation of a process,” with the process being the conceptualization in the given context that led to how the expression was formulated. This can be more or less elaborate (more or less schematic and metonymic) for any verbal or gestural expression.

In terms of limitations of the study, we acknowledge that the setting was not completely authentic. It was not a live lecture being interpreted for an audience that was visibly present. This was a factor of wanting to have the interpreters only hear the lectures without being influenced by seeing the original speakers’ gestures; there was also the logistical factor of wanting several dozen interpreters to interpret the same lecture and the logistical and scheduling challenges that would have arisen if we had had to bring in an audience for each interpreting session. An extension of this project will have interpreters view the video-recordings of the lectures that they are interpreting, bringing the study closer to authentic conditions, particularly those used for interpreting in videoconferences. The study also faced difficulties arising from the COVID-19 pandemic, namely that the lockdown restrictions meant that interpreters could only participate during certain time periods. This resulted in a somewhat larger number of participants for the Russian-English study than for the Russian-German study, as the data collection for the latter overlapped with the pandemic.

In conclusion, we see that the difference in status between the semiotic systems of lexico-grammar versus manual gesture plays out in terms of the difference in how principal footing can be attributed on the verbal level in simultaneous interpreting and in terms of the use of gesture. Looking at gesture, we see in many cases the lamination and the ambiguity of the principal footing. This schematicity and ambiguity of gesture may be part and parcel of what is involved in interpreters’ thinking for speaking. In particular, their frequent use of pragmatic gestures plays on the border between what the original lecturer may have done when expressing a stance toward the topic mentioned verbally and the interpreter’s own stance.

McNeill (2000, 2013) argues that gesture provides a window onto the mind. Through the window of simultaneous interpreters’ gestures, we can catch glimpses into the blending of viewpoints that thinking for simultaneous interpreting appears to involve (as per Cienki and Iriskhanova, 2020) in different ways, changing over time. Such thinking for interpreting clearly differs from the process of unpacking one’s own idea units that McNeill argues takes place in spontaneously expressing one’s own thoughts. The ideas to be spoken are presented to interpreters in the utterances in the source language, rather than arising from their own personal engagement in thinking and interacting with others. As a re-presenter of the original lecturer’s words in another language, interpreters might project what such a lecturer might have done in the context of presenting the given ideas, but they also surely incorporate elements of their own repertoire of how they speak and present ideas, including gesturally.

## Data availability statement

The raw data supporting the conclusions of this article will be made available by the authors, without undue reservation.

## Ethics statement

The studies involving humans were approved by the Research Ethics Committee of the Science and Technology Board at Moscow State Linguistic University. The studies were conducted in accordance with the local legislation and institutional requirements. The participants provided their written informed consent to participate in this study. Written informed consent was obtained from the individual(s) for the publication of any potentially identifiable images or data included in this article.

## Author contributions

AC: Conceptualization, Methodology, Supervision, Writing – original draft, Writing – review & editing.

## Footnotes

^1^“Существует ли сегодня угроза шестого массового вымирания видов?” https://postnauka.ru/video/49851, lecturer: Nikolai Dronin.

^2^https://www.ted.com/talks/michael_benton_mass_extinctions_and_the_future_of_life_on_earth?language=en, lecturer: Michael Benton.

^3^“Das Ende der Evolution” https://www.ardmediathek.de/video/tele-akademie/prof-dr-matthias-glaubrecht-das-ende-der-evolution/swr/Y3JpZDovL3N3ci5kZS9hZXgvbzEyMDkzOTk/, lecturer: Matthias Glaubrecht.

^4^The first-person plural pronoun ‘we’ will be used as the research was conducted in collaboration with members of the lab listed in the Acknowledgments.

^5^From the Max Planck Institute for Psycholinguistics, The Language Archive, Nijmegen, The Netherlands. https://archive.mpi.nl/tla/elan.

^6^The American English spelling ‘adapter’ is used here, but note (if searching the literature) that much research on them employs the British English spelling ‘adaptor.’

^7^See, for example, the well-known image of the dodo painted by George Edwards in 1626. https://en.wikipedia.org/wiki/Dodo#Contemporary_depictions.

^8^The interpreter then makes additional pragmatic gestures for emphasis during this utterance, not indicated here.
